# Learning curves in laparoscopic distal pancreatectomy: a different experience for each generation

**DOI:** 10.1097/JS9.0000000000000408

**Published:** 2023-05-05

**Authors:** Tess M.E. van Ramshorst, Bjørn Edwin, Ho-Seong Han, Masafumi Nakamura, Yoo-Seok Yoon, Takao Ohtsuka, Tore Tholfsen, Marc G. Besselink, Mohammad Abu Hilal

**Affiliations:** a Department of General Surgery, Istituto Ospedaliero Fondazione Poliambulanza, Brescia, Italy; b Amsterdam UMC, University of Amsterdam, Department of Surgery; c Cancer Center Amsterdam, Amsterdam, The Netherlands; d The Intervention Centre and Department of HPB Surgery, Oslo University Hospital, also Institute of Medicine, University of Oslo; e Department of Hepato-Pancreato-Biliary Surgery, Rikshospitalet, Oslo University Hospital, Oslo, Norway; f Department of Surgery and Oncology, Graduate School of Medical Sciences, Kyushu University, Fukuoka; g First Department of Surgery, Kagoshima University School of Medicine, Kagoshima, Japan; h Department of Surgery, Seoul National University Bundang Hospital, Seoul National University College of Medicine, Seoul, Republic of Korea; i Department of Surgery, University Hospital Southampton National Health Service, Southampton, Hampshire, United Kingdom

**Keywords:** different generations, laparoscopic distal pancreatectomy, learning curve, outcomes, risk-adjusted cumulative sum analysis

## Abstract

Learning curves of laparoscopic distal pancreatectomy (LDP) are mostly based on ‘self-taught’ surgeons who acquired sufficient proficiency largely through self-teaching. No learning curves have been investigated for ‘trained’ surgeons who received training and built on the experience of the ‘self-taught’ surgeons. This study compared the learning curves and outcome of LDP between ‘self-taught’ and ‘trained’ surgeons in terms of feasibility and proficiency using short-term outcomes.

**Materials and methods:**

Data of consecutive patients with benign or malignant disease of the left pancreas who underwent LDP by four ‘self-taught’ and four ‘trained’ surgeons between 1997 and 2019 were collected, starting from the first patient operated by a contributing surgeon. Risk-adjusted cumulative sum (RA-CUSUM) analyses were performed to determine phase-1 feasibility (operative time) and phase-2 proficiency (major complications) learning curves. Outcomes were compared based on the inflection points of the learning curves.

**Results:**

The inflection points for the feasibility and proficiency learning curves were 24 and 36 procedures for ‘trained’ surgeons compared to 64 and 85 procedures for ‘self-taught’ surgeons, respectively. In ‘trained’ surgeons, operative time was reduced after completion of the learning curves (230.5–203 min, *P=*0.028). In ‘self-taught’ surgeons, operative time (240–195 min, *P*≤0.001), major complications (20.6–7.8%, *P=*0.008), and length of hospital stay (9–5 days, *P*≤0.001) reduced after completion of the learning curves.

**Conclusion:**

This retrospective international cohort study showed that the feasibility and proficiency learning curves for LDP of ‘trained’ surgeons were at least halved as compared to ‘self-taught’ surgeons.

## Graphical abstract

**Figure:**
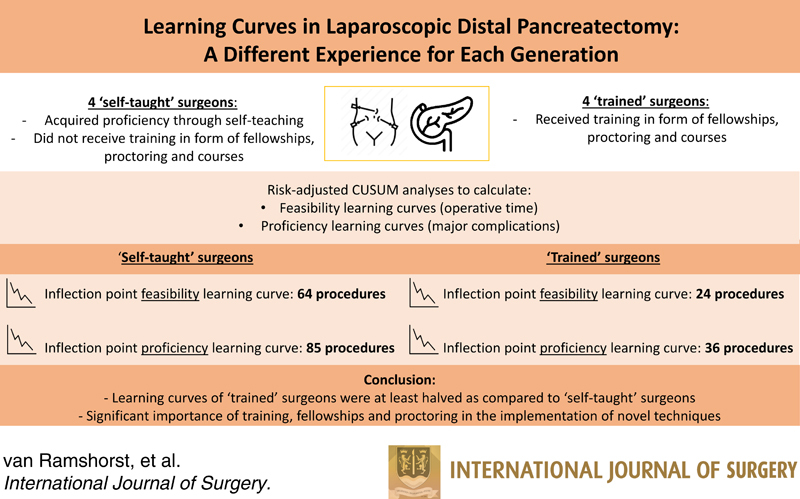

## Introduction

HIGHLIGHTS

- Feasibility learning curve ‘trained’ surgeons 24 versus 64 cases of ‘self-taught’ surgeons.
- Proficiency learning curve ‘trained’ surgeons 36 versus 85 cases of ‘self-taught’ surgeons.
- Learning curves of ‘trained’ surgeons are at least halved as compared to ‘self-taught’ surgeons.
- Training and proctoring lead to a significant reduction in learning curves.
- Training and proctoring lead to early improvements in surgical outcomes.

The implementation of laparoscopic distal pancreatectomy (LDP) has developed exponentially since its introduction in 1994^1,2^. In recent years, numerous observational studies and systematic reviews comparing LDP with open distal pancreatectomy (ODP) demonstrated comparable or favorable outcomes of LDP, making LDP considered a safe alternative to ODP^3–8^. Two randomized trials showed the superiority of LDP over ODP in terms of time to functional recovery, length of hospital stay, intraoperative blood loss, and delayed gastric emptying with comparable mortality rates^9,10^. Based on these promising results, the Miami Guidelines on minimally invasive pancreatic resection recommended LDP over ODP as surgical treatment for benign and low-grade malignant tumors in experienced centers^11^.

However, as LDP remains a complex and technically difficult procedure, careful implementation is required to minimize a negative impact of the learning curve on patient outcomes. For that reason, the implementation of LDP has followed the surgical IDEAL framework, a structured model that describes five stages of innovation in surgery to ensure the safe implementation of novel surgical techniques^12–15^. Besides this, increasing attention has been devoted to the assessment of learning curves since studies have shown that the adoption of minimally invasive pancreatic surgery during the learning curve can lead to an increase in morbidity and mortality rates^16,17^. As a result, many studies assessing the learning curve of laparoscopic distal pancreatectomy have been published^18–22^. However, most of these studies investigated the learning curves in single-center case series or in the experience of the first generation of ‘self-taught’ surgeons who acquired sufficient proficiency largely through self-teaching, while studies to the learning curves of the next generation of ‘trained’ surgeons are still lacking.

Therefore, the aim of this study is to compare the learning curves and outcome of LDP in ‘self-taught’ surgeons and ‘trained’ surgeons in terms of phase-1 feasibility and phase-2 proficiency using intraoperative and postoperative outcomes such as operative time and major complications. This may eventually contribute to a more nuanced learning curve identification specifically targeted at different generations of surgeons.

## Methods

### Patients and design

Data of all consecutive patients who underwent elective LDP by ‘self-taught’ surgeons and ‘trained’ surgeons in the period 1997–2019 were collected from a retrospective database of tertiary referral centers participating in the European Consortium on Minimally Invasive Pancreatic Surgery (E-MIPS) or International Consortium on Minimally Invasive Pancreatic Surgery (I-MIPS). The collected data included the name of the surgeon so inclusion criteria for participating surgeons could be determined. Surgeons who were experienced with a minimum of 40 LDPs in total with at least 10 LDPs per year and who performed their LDPs in the same tertiary referral institution (i.e. no outside institutions) were included. The study was conducted according to the principles of the Declaration of Helsinki (64th Fortaleza Brazil, October 2013) and followed the guidelines of the Strengthening The Reporting Of Cohort Studies in Surgery (STROCSS, Supplemental Digital Content 2, http://links.lww.com/JS9/A436)^23^. The ethical board of Amsterdam UMC waived the need for informed consent due to the retrospective design.

### Outcomes and definitions

Surgeons were labeled as ‘self-taught’ surgeons if they independently started performing LDP during time phase-2 of the IDEAL framework, the Development and Exploration phase^15^, without receiving any specific form of training. The next generation of ‘trained’ surgeons were defined as fellowship-trained surgeons who received training without prior independent experience in LDP^24^. They received education in LDP and had training possibilities in the forms of fellowships, simulation and proctored programs, and (hands-on) courses. Preoperative variables and operative details included baseline characteristics such as age, sex, and BMI, American Society of Anesthesiologists (ASA) classification^25^, tumor size, pancreatic ductal adenocarcinoma (PDAC), and the type of spleen-preserving procedure. Study endpoints included surgical variables such as operative time, intraoperative blood loss, multivisceral resection, conversion, and failure to preserve the spleen and postoperative variables as clinically relevant postoperative pancreatic fistula (POPF grade B/C), major complications, length of hospital stay, readmission, and mortality. Postoperative outcomes were recorded up to 90 days postoperatively. The definition of clinically relevant pancreatic fistula followed the definitions of the International Study Group on Pancreatic Surgery (ISGPS)^26^. Major complications were classified by the Clavien–Dindo classification of surgical complications, defined as a Clavien–Dindo grade 3a or higher^27^. Conversion refers to any procedure that started as a laparoscopic procedure but required conversion to open surgery^28^. Spleen-preserving procedures were classified according to the Kimura^29^ splenic vessels preserving or Warshaw^30^ splenic vessels resecting method. Learning curves for feasibility (phase-1) were based on operative time, and learning curves for proficiency (phase-2) on major complications, as described in previous literature^18,31^.

### Statistical analysis

Data analysis was performed using IBM SPSS statistics for Windows version 26.0 (IBM). Categorical data were presented as proportions and compared between groups using *χ* ^2^ test. Continuous data were reported as median with interquartile range (IQR) due to the skewness of the data. The Mann–Whitney *U* test was used to compare the continuous data between two groups, and the Kruskal–Wallis test was used for comparison between more than two groups. Univariable and multivariable linear and logistic regression analyses were performed to create risk-adjusted models for variables independently associated with operative time and major complications. Variables with *P*<0.1 at univariable analysis were included in the multivariable model. Statistical significance was set at a two-sided *P* value of <0.05.

#### Learning curve analysis

Risk-adjusted cumulative sum (RA-CUSUM) analysis was used to establish feasibility and proficiency learning curves. RA-CUSUM analysis is a graphical method that portrays the accumulated sequential difference between the data of individual cases (observed outcome) and the mean value of all data (expected outcome) while adjusting for the risk of a particular case mix^32,33^. To establish learning curves for the groups of either ‘self-taught’ or ‘trained’ surgeons rather than for 8 individual surgeons, the methodology of a similar study by Halls *et al*.^34^ was followed, although there are also examples of other recent publications that performed multi-surgeon or multicenter CUSUM analysis^35,36^. First, data from all patients were combined to construct risk-adjusted regression models for operative time and major complications using univariable and multivariable regression analyses with backward selection. The final model of operative time included male sex, ASA I–II classification, and multivisceral resection, and the final model of major complications included only BMI >30 kg/m^2^ (Supplementary Tables 1 and 2, Supplemental Digital Content 1, http://links.lww.com/JS9/A435). After this, data of ‘self-taught’ surgeons and ‘trained’ surgeons were analyzed separately to create separate RA-CUSUM plots. The RA-CUSUM plots of the operative time and major complications were calculated using the following equation: RA-CUSUM = $\sum_{i-1}^{n}\left(X_{i}-\tau \right)+{\left(\;-\;1\right)}^{x}P_{i}$ . $X_{i}$ indicates the observed operative time or occurrence of major complications, τ indicates the mean operative time or event rate of major complications. $P_{i}$ indicates the expected mean operative time or event rate of major complications based on the logistic regression model in each case. The cases of each surgeon within the group of ‘self-taught’ or ‘trained’ surgeons were chronologically numbered from the first case to the last case based on the date of operation. Hereafter, the corresponding cases (i.e. case 1 with case 1, case 2 with case 2, and so on) of each surgeon within a group were pooled with equal weighting. In this way, a specific case point represented the average performance of a group (consisting of four ‘self-taught’ or four ‘trained’ surgeons) instead of an individual surgeon. Since participating surgeons only had to perform a minimum of 40 LDPs to be included in the study, not all surgeons performed the same number of LDPs, which may have meant that in higher case numbers, the learning curve point was based on fewer surgeons than four. In the final RA-CUSUM plot, every case was plotted from left to right, where a rising curve indicated longer operative times or higher occurrence of complications compared to the overall mean, whereas a falling curve indicated shorter operative times or lower complications compared to the overall mean thus indicating surgical success. A plateau in the learning curve indicated a steady performance level. Changes in performances or in learning curve phases were based on the inflection points in the curves. The significance of these points was tested by comparing the clinical outcomes of either ‘self-taught’ surgeons or ‘trained’ surgeons before, between, and after the inflection points of the feasibility and proficiency learning curves.

## Results

### Study population

In total, 639 patients undergoing LDP by four ‘trained’ surgeons and four ‘self-taught’ surgeons from five centers were included. Overall, 215 patients were operated by ‘trained’ surgeons and 424 by ‘self-taught’ surgeons. Patient characteristics and operative details are shown in Table 1. Overall, ‘trained’ surgeons performed fewer Kimura spleen-preserving procedures (21.3 vs. 83.6%, *P*≤0.001) compared to ‘self-taught’ surgeons. The median operative times were 210 and 215 min (*P*=0.769), median blood loss was 100 and 200 ml (*P*≤0.001), and the rate of conversion was 4.2 and 4.2% (*P=*0.972) for ‘trained’ surgeons and ‘self-taught’ surgeons, respectively.

**Table 1:** Patients characteristics and operative details of all procedures performed by ‘trained’ surgeons and ‘self-taught’ surgeons.

Characteristics	‘Trained’ surgeons (*n*=215)	‘Self-taught’ surgeons (*n*=424)	*P*
Age, years, median (IQR)	64 (47–72)	60 (44–70)	0.055
Male, *n* (%)	98 (45.6)	157 (37.0)	0.037
BMI, kg/m^2^, median (IQR)	24.3 (21.7–27.2)	24.3 (21.2–27)	0.437
BMI>30 kg/m^2^, *n* (%)	27 (12.6)	37 (11.3)	0.652
ASA score 1–2, *n* (%)	176 (82.2)	360 (84.9)	0.386
Previous abdominal surgery, *n* (%)	78 (36.3)	124 (406)	0.147
Tumor size, mm, median (IQR)	29 (19–40)	30 (20–45)	0.079
PDAC, *n* (%)	34 (15.8)	56 (13.2)	0.337
Splenectomy, *n* (%)	122 (56.7)	264 (62.3)	0.178
Kimura technique in spleen-preserving distal pancreatectomy, *n* (%)	19 (21.3)	133 (83.6)	<0.001
Operative time, min, median (IQR)	210 (156–280)	215 (160–275)	0.769
Blood loss, ml, median (IQR)	100 (50–242.5)	200 (60.8–400)	<0.001
Conversion, *n* (%)	9 (4.2)	18 (4.2)	0.972

Values in parentheses are percentages unless mentioned otherwise. Percentages may not add up due to rounding and missing data.

ASA, American Society of Anesthesiologists; IQR, interquartile range; PDAC, pancreatic ductal adenocarcinoma.

### Learning curve and inflection points

**Figure 1:**
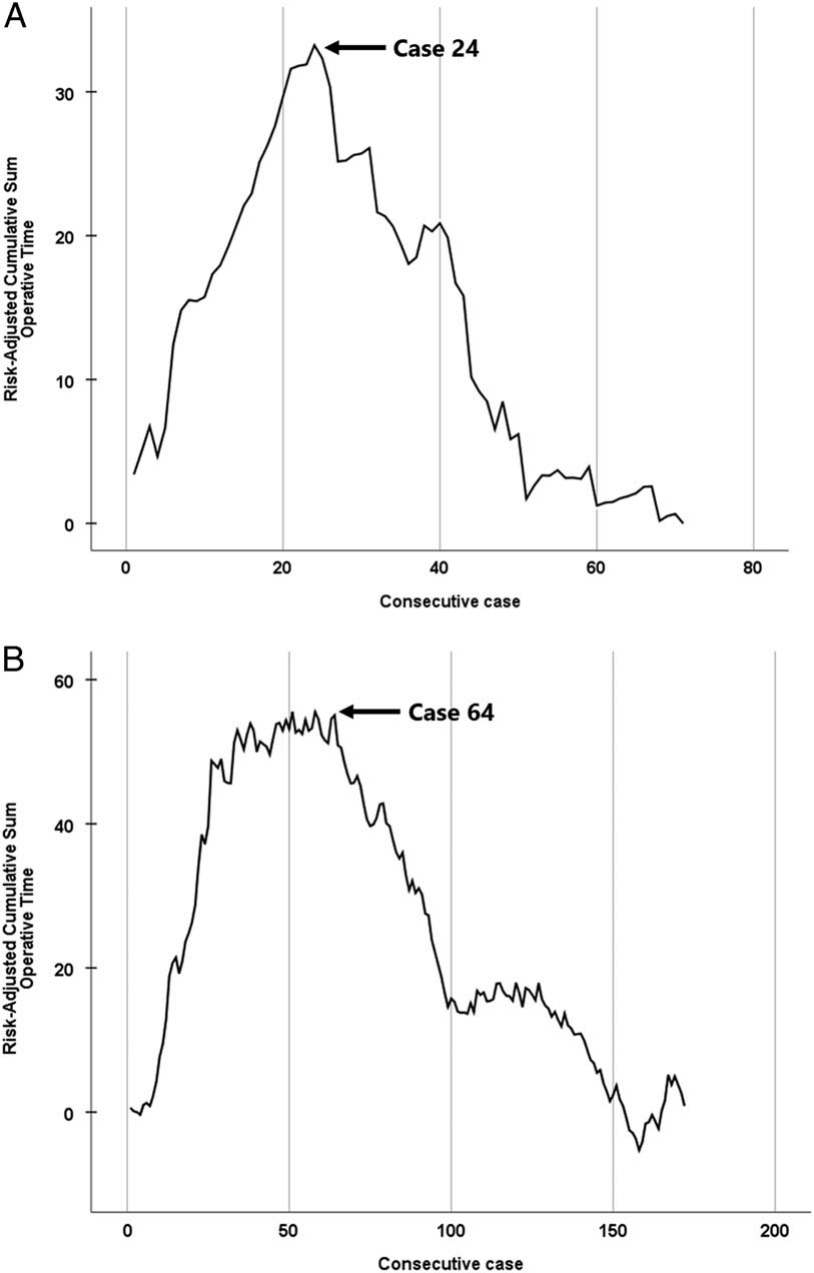
RA-CUSUM (risk-adjusted cumulative sum) phase-1 feasibility learning curves for operative time: (A) among ‘trained’ surgeons and (B) among ‘self-taught’ surgeons. (A) ‘Trained’ surgeons; an inflection point is observed in case 24. (B) ‘Self-taught’ surgeons; an inflection point is observed in case 64.

**Figure 2:**
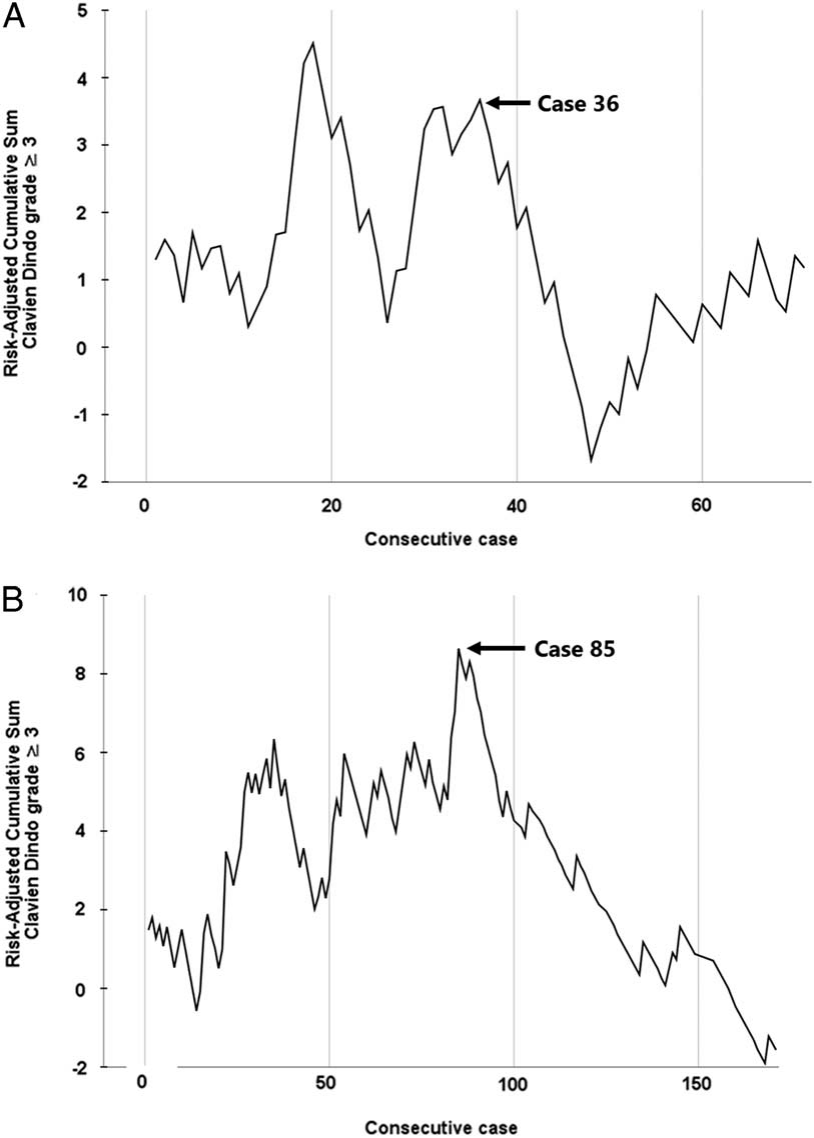
RA-CUSUM (risk-adjusted cumulative sum) phase-2 proficiency learning curves for major complications: (A) among ‘trained’ surgeons and (B) among ‘self-taught’ surgeons. (A) ‘Trained’ surgeons; an inflection point is observed in case 36. (B) ‘Self-taught’ surgeons; an inflection point is observed in case 85.

Analysis of the RA-CUSUM learning curves of the ‘trained’ surgeons revealed inflection points of the feasibility learning curve for an operative time at 24 procedures (Fig. 1A) and the proficiency learning curve for major complications at 36 procedures (Fig. 2A). Learning curves of the ‘self-taught’ surgeons showed inflection points at 64 and 85 procedures, respectively (Figs 1B, 2B). The learning curves are more detailed and displayed in Supplementary Figures 1 and 2, Supplemental Digital Content 1, http://links.lww.com/JS9/A435.

### Outcomes within ‘trained’ surgeons

Analysis of the outcomes and operative trends in ‘trained’ surgeons based on the inflection points of 24 (feasibility) and 36 (proficiency) procedures are shown in Table 2. A significant increase in ASA III–IV patients was found in the proficiency phase compared to the feasibility phase (9.5 vs. 25%, *P=*0.018). Operative time significantly reduced after the feasibility learning curve (230.5–187.5 min, *P=*0.028). Intraoperative blood loss decreased as well after the feasibility learning curve but increased after the proficiency learning curve, so no significance was reached (120–71.5–100 ml, *P=*0.532). The rate of major complications increased during the proficiency phase but decreased after its completion (25.5–17.4%, *P=*0.480). Failure rates of spleen preservation significantly increased during the learning curve phases (2–21.6%, *P=*0.011).

**Table 2:** Patient, tumor, and surgical characteristics of LDPs by ‘trained’ surgeons based on the inflection points on RA-CUSUM analysis.

Characteristics	0–24 LDP (*n*=96)	25–36 LDP (*n*=48)	After 36 LDP (*n*=71)	*P*
Age, years, median (IQR)	62 (41.3–72)	64.5 (52–71)	65 (52–72)	0.446
Male, *n* (%)	40 (41.7)	30 (62.5)	28 (39.4)	0.027
BMI, kg/m^2^, median (IQR)	24 (21.1–26.4)	23.9 (21.7–28.2)	24.9 (22–27.2)	0.541
BMI>30 kg/m^2^, *n* (%)	10 (10.4)	8 (16.7)	9 (12.7)	0.566
ASA score 1–2, *n* (%)	86 (90.5)	36 (75.0)	54 (76.1)	0.018
Previous abdominal surgery, *n* (%)	29 (30.2)	20 (41.7)	29 (40.8)	0.250
Tumor size, mm, median (IQR)	29 (20–37.8)	28 (15.8–38.5)	30 (19–40.5)	0.761
PDAC, *n* (%)	14 (14.6)	8 (16.7)	12 (16.9)	0.906
Splenectomy, *n* (%)	46 (47.9)	34 (70.8)	42 (59.2)	0.029
Blood loss, ml, median (IQR)	120 (50–300)	71.5 (50–200)	100 (50–200)	0.532
Multivisceral resection, *n* (%)	2 (2.1)	2 (4.2)	7 (9.9)	0.074
Conversion, *n* (%)	3 (3.1)	2 (4.2)	4 (5.6)	0.726
Operative time, min, median (IQR)	230.5 (173–319.5)	187.5 (150.5–252.8)	203 (152–266)	0.028
Pancreatic fistula grade B/C, *n* (%)	19 (19.8)	13 (27.1)	15 (21.4)	0.604
Clavien–Dindo grade ≥3, *n* (%)	21 (22.6)	12 (25.5)	12 (17.4)	0.480
Grade 1–2	30	14	23	
Grade 3	18	11	11	
Grade 4	1	1	1	
Grade 5	2	0	0	
Failure to preserve the spleen, *n* (%)	1 (2)	3 (17.6)	8 (21.6)	0.011
Length of hospital stay, days, median (IQR)	7 (5–12)	6.5 (5–12)	6 (4–10)	0.324
Readmission <90 days, *n* (%)	12 (12.5)	4 (8.3)	13 (18.3)	0.274
Mortality <90 days, *n* (%)	2 (2.1)	0 (0)	0 (0)	0.286

Values in parentheses are percentages unless mentioned otherwise. Percentages may not add up due to rounding and missing data.

ASA, American Society of Anesthesiologists; IQR, interquartile range; LDP, laparoscopic distal pancreatectomy; PDAC, pancreatic ductal adenocarcinoma, RA-CUSUM, risk-adjusted cumulative sum.

### Outcomes within ‘self-taught’ surgeons

Analysis of the outcomes and operative trends of the ‘self-taught’ surgeons based on the inflection points of 64 (feasibility) and 85 (proficiency) cases are shown in Table 3. Through the learning curve phases, ‘self-taught’ surgeons performed significantly more resections for PDACs (6.1–25.9%, *P*≤0.001) and treated older and more obese patients (54.5–68 years, *P*≤0.001 and 23.4–25.6 kg/m^2^, *P*≤0.001, respectively). Operative time and intraoperative blood loss significantly reduced after the feasibility learning curve (240–195 min, *P*≤0.001 and 200–150 ml, *P=*0.019). The rate of major complications remained unchanged during the feasibility and proficiency phases but significantly decreased after surpassing them (20.6–7.8%, *P=*0.008). Length of hospital stay significantly decreased during and after both learning curve phases (9–6–5 days, *P*≤0.001). These changes were associated with a slightly decreased readmission rate during the feasibility and proficiency phases (8.2–6.3%) but a significantly higher readmission rate after their completion (6.3–19.8%, *P=*0.002).

**Table 3:** Patient, tumor, and surgical characteristics of LDP by ‘self-taught’ surgeons based on the inflection points on RA-CUSUM analysis.

Characteristics	0–64 LDP (*n*=244)	65–85 LDP (*n*=63)	After 85 LDP (*n*=117)	*P*
Age, years, median (IQR)	54.5 (40–66.8)	59 (41–69)	68 (60–72)	<0.001
Male, *n* (%)	88 (36.1)	22 (34.9)	70 (40.2)	0.700
BMI, kg/m^2^, median (IQR)	23.4 (20.3–26.1)	24.2 (21–26.8)	25.6 (23.1–29.1)	<0.001
BMI>30 kg/m^2^, *n* (%)	10 (5.4)	5 (11.4)	22 (22.4)	<0.001
ASA score 1–2, *n* (%)	209 (86.0)	54 (84.4)	97 (82.9)	0.737
Previous abdominal surgery, *n* (%)	69 (30.3)	22 (34.9)	33 (28.7)	0.683
Tumor size, mm, median (IQR)	32 (20 – 50)	32 (20.3–40.8)	30 (21.5–45)	0.899
PDAC, *n* (%)	15 (6.1)	11 (17.5)	30 (25.9)	<0.001
Splenectomy, *n* (%)	129 (53.1)	42 (65.5)	93 (79.5)	<0.001
Blood loss, ml, median (IQR)	200 (70.5–400)	150 (40–300)	200 (80–400)	0.019
Multivisceral resection, *n* (%)	17 (7)	8 (12.9)	12 (10.3)	0.267
Conversion, *n* (%)	10 (4.1)	1 (1.6)	7 (6)	0.372
Operative time, min, median (IQR)	240 (180–305)	195 (145–240)	195 (149–240)	<0.001
Pancreatic fistula grade B/C, *n* (%)	42 (17.4)	7 (11.1)	19 (16.4)	0.485
Clavien–Dindo grade ≥3, *n* (%)	50 (20.6)	13 (20.6)	9 (7.8)	0.008
Grade 1–2	48	15	48	
Grade 3	46	13	5	
Grade 4	4	0	2	
Grade 5	0	0	2	
Failure to preserve the spleen, *n* (%)	4 (3.4)	0 (0)	3 (11.1)	0.114
Length of hospital stay, days, median (IQR)	9 (6–13)	6 (4–8)	5 (4–7)	<0.001
Readmission <90 days, *n* (%)	20 (8.2)	4 (6.3)	23 (19.8)	0.002
Mortality <90 days, *n* (%)	1 (0.6)	0 (0)	2 (2.3)	0.312

Values in parentheses are percentages unless mentioned otherwise. Percentages may not add up due to rounding and missing data.

ASA, American Society of Anesthesiologists; IQR, interquartile range; LDP, laparoscopic distal pancreatectomy; PDAC, pancreatic ductal adenocarcinoma; RA-CUSUM, risk-adjusted cumulative sum.

### ‘Trained’ surgeons vs. ‘self-taught’ surgeons based on inflection points

Patients’ age, BMI, and the proportion of PDAC were higher in the feasibility phase of ‘trained’ surgeons compared to the feasibility phase of the ‘self-taught’ surgeons (62 vs. 54.5 years, 24 vs. 23.4 kg/m^2^, and 14.6 vs. 6.1%, respectively). Patients in the feasibility phase of the ‘trained’ surgeons were, in this regard, more comparable to patients in the proficiency phase of the ‘self-taught’ surgeons (62 vs. 59 years, 24 vs. 24.2 kg/m^2^, and 14.6 vs. 17.5%, respectively). In contrast, ‘self-taught’ surgeons operated larger tumors and performed more multivisceral resections in comparison to ‘trained’ surgeons in the feasibility phase (32 vs. 29 mm, 7 vs. 2.1%, respectively).

## Discussion

This retrospective international cohort study found that the feasibility (24 vs. 64) and proficiency (36 vs. 85) learning curves for LDP were at least halved among ‘trained’ surgeons as compared to ‘self-taught’ surgeons. Intraoperative parameters such as operative time and blood loss, as well as postoperative outcomes such as major complications and length of hospital stay, improved for both groups after the learning curves.

Previous studies have identified learning curves for LDP ranging from 10 to 50 procedures^18–22,31,37^. In those studies, intraoperative parameters such as operative time and blood loss were used to assess the learning curves. These parameters are assumed to illustrate the surgeons’ ability to complete the procedure and are therefore used to reflect the feasibility and first phase of the learning curve^18,31^. The feasibility learning curves of 24 procedures for ‘trained’ surgeons versus 64 procedures for ‘self-taught’ surgeons in the present study were based on operative time and did therefore align with the previously published learning curves. On the contrary, the obtained proficiency learning curve of 85 procedures of ‘self-taught’ surgeons in this study is substantially longer. However, proficiency learning curves reflect more than just a surgeon’s ability to complete the procedure; they also reflect surgical proficiency and standards, thus the surgeon’s ability to achieve excellence in surgical outcomes. This should be measured by postoperative outcomes such as major complications, readmissions, or length of hospital stay, which indicate advanced experience and thus reflect the second phase of the learning curve^31^. Logically, it takes more procedures to reach this phase. A previous study has suggested that this can be reached between cases 72 and 100, most probably around 80 LDP procedures, based on readmissions and length of hospital stay^18^. The proficiency learning curves of this study were based on major complications and confirmed the previous findings, suggesting that it can require 85 procedures to overcome the proficiency learning curve phase.

For both ‘self-taught’ and ‘trained’ surgeons, operative trends and clinical outcomes improved after the inflection points of the feasibility and proficiency learning curves. With increasing experience, ‘self-taught’ surgeons operated on older and more complex patients while achieving a significant decrease in intraoperative blood loss, operative time, major complications, and length of hospital stay. Although the latter (shorter hospital stay) may have been at the cost of an increase in readmissions. With respect to the ‘trained’ surgeons, only a significant improvement in operative time was found during the feasibility and proficiency phase, but many operative trends were observed. With increasing experience, ‘trained’ surgeons operated on older patients with higher ASA scores, a higher proportion of PDAC, and more multivisceral resections. However, this may also have contributed to the slight increase in operative time after the feasibility and proficiency phases. Postoperative outcomes such as POPF grade B/C and major complications substantially decreased after the learning curve phases but did not reach statistical significance.

An important finding, and consistent with the findings of a comparative study in laparoscopic liver surgery^34^, is that the learning curves of ‘trained’ surgeons of the present study were shorter than those of ‘self-taught’ surgeons. Those findings suggest that ‘trained’ surgeons may overcome their learning curves much faster than ‘self-taught’ surgeons may. Since the introduction of LDP in 1994^1^, its implementation has followed the steps of the IDEAL framework for surgical innovation. ‘Self-taught’ surgeons of the present study started performing LDP in 1997 when LDP was still in its development and exploration phase. In this era, they had no prior knowledge of minimally invasive pancreatic surgery, consensus guidelines, innovation, or support for a specific technique or instrument. Therefore, ‘self-taught’ surgeons had to find their way with the complexity and novelty of the laparoscopic technique, which was mainly based on the concept of ‘trial and error’. ‘Trained’ surgeons, on the other hand, started performing LDP in 2012. Meanwhile, laparoscopic surgery had become more widely applied, studies on surgical techniques had been published, guidelines had been introduced, and instruments had been improved. Moreover, ‘trained’ surgeons presumably thrived on the experiences gained by the ‘self-taught’ surgeons and benefited from proctoring, fellowships and training possibilities. The shortened learning curves of ‘trained’ surgeons compared to ‘self-taught’ surgeons of the present study do, therefore, not only reflect a different generation but also the effect of a different era centered on technical innovation, improved knowledge, evidence-based practice, and training possibilities. It illustrates that learning curves should not be considered as defined aspects but as flexible concepts that are subject to many external circumstances.

Several limitations apply to this study. First and most importantly, its retrospective design. Due to the main objective to examine learning curves from different time stages in the surgical innovation of LDP, the time aspect of the study has a potential impact in which changes in preoperative and postoperative policies may have influenced the outcomes. Interestingly, in the risk-adjusted regression model, ASA 1–2 was associated with increased operative time, which is a finding we are unable to scientifically explain but may be due to the retrospective data collection. In addition to this, as the application of laparoscopic surgery increased over time with widened indications for LDP, ‘trained’ surgeons, as compared to ‘self-taught’ surgeons, probably performed more cases in the same time period. This also applies to other laparoscopic procedures and might have contributed to the reduction of the learning curve of ‘trained’ surgeons. Second, no data was available on the surgeons’ level of experience in open distal pancreatectomy. This could be of interest as it may have positively affected their LDP performance. Third, as LDP evolves through time, just like any other surgical procedure, the obtained learning curves of this study are not static and must be revised periodically in relation to the future adoption of LDP.

The main strength of this study is that it is the first multicenter study to identify and compare learning curves in LDP for different generations of surgeons as well as different surgical learning curve phases. Many studies on the learning curves in LDP date back to earlier years and investigated the learning curves of surgeons of the first generation, while this study aimed to examine the effect of further implementation and training in LDP on the second generation of surgeons. The obtained differences in learning curves of this study imply that it should be recommended that surgeons of the current generation must first complete acknowledged training programs in terms of fellowship, proctored programs, or courses before they start performing LDP. Additionally, the obtained learning curves may be adopted in the design of future surgical trials.

## Conclusion

This multicenter study shows the impact of the implementation and standardization of LDP over the last two decades and illustrates that learning curves must be considered flexible curves that are dependent on many factors, including time, innovation, evidence-based practice, and training. The reduction in the learning curve of ‘trained’ surgeons compared to ‘self-taught’ surgeons show the importance of education and training in the implementation of novel techniques and that these elements can contribute to early improvements of intra and postoperative outcomes.

## Ethical approval

The ethical board of Amsterdam UMC approved this study and waived the need for informed consent due to the retrospective design and the use of pseudonymized data.

## Sources of funding

This research did not receive any specific grant from funding agencies in the public, commercial, or not-for-profit sectors.

## Author contribution

T.M.E.v.R.: study conception and design, analysis and interpretation of data, drafting of the manuscript, and final approval; B.E., H.-S.H., M.N., Y.-S.Y., T.O., and T.T.: acquisition of the data, critical revision, and final approval; M.G.B.: study conception and design, acquisition of data, critical revision, and final approval; M.A.H.: study conception and design, acquisition of data, drafting of the manuscript, and final approval.

## Conflicts of interest disclosure

There are no conflicts of interest.

## Research registration unique identifying number (UIN)

1. Name of the registry: https://www.clinicaltrials.gov/
2. Unique identifying number or registration ID: NCT05595343.
3. Hyperlink to your specific registration (must be publicly accessible and will be checked): https://clinicaltrials.gov/ct2/show/NCT05595343

## Guarantor

Tess M.E. van Ramshorst and Mohammad Abu Hilal.

## Data availability statement

The data are confidential and only available upon reasonable request.

## Supplementary Material

## References

[1] Cuschieri A Jakimowicz JJ van Spreeuwel J . Laparoscopic distal 70% pancreatectomy and splenectomy for chronic pancreatitis. Ann Surg 1996;223:280–285. 8604908 10.1097/00000658-199603000-00008 PMC1235116

[2] Abu Hilal M Takhar AS . Laparoscopic left pancreatectomy: current concepts. Pancreatology 2013;13:443–448. 23890145 10.1016/j.pan.2013.04.196

[3] de Rooij T Jilesen AP Boerma D . A nationwide comparison of laparoscopic and open distal pancreatectomy for benign and malignant disease. J Am Coll Surg 2015;220:263–70.e1. 25600974 10.1016/j.jamcollsurg.2014.11.010

[4] Jusoh AC Ammori BJ . Laparoscopic versus open distal pancreatectomy: a systematic review of comparative studies. Surg Endosc 2012;26:904–913. 22083328 10.1007/s00464-011-2016-3

[5] Mehrabi A Hafezi M Arvin J . A systematic review and meta-analysis of laparoscopic versus open distal pancreatectomy for benign and malignant lesions of the pancreas: it’s time to randomize. Surgery 2015;157:45–55. 25482464 10.1016/j.surg.2014.06.081

[6] Riviere D Gurusamy KS Kooby DA . Laparoscopic versus open distal pancreatectomy for pancreatic cancer. Cochrane Database Syst Rev 2016;4:CD011391. 27043078 10.1002/14651858.CD011391.pub2 PMC7083263

[7] Sui CJ Li B Yang JM . Laparoscopic versus open distal pancreatectomy: a meta-analysis. Asian J Surg 2012;35:1–8. 22726557 10.1016/j.asjsur.2012.04.001

[8] Abu Hilal M Hamdan M Di Fabio F . Laparoscopic versus open distal pancreatectomy: a clinical and cost-effectiveness study. Surg Endosc 2012;26:1670–1674. 22179475 10.1007/s00464-011-2090-6

[9] Bjornsson B Larsson AL Hjalmarsson C . Comparison of the duration of hospital stay after laparoscopic or open distal pancreatectomy: randomized controlled trial. Br J Surg 2020;107:1281–1288. 32259297 10.1002/bjs.11554

[10] de Rooij T van Hilst J van Santvoort H . Minimally invasive versus open distal pancreatectomy (LEOPARD): a multicenter patient-blinded randomized controlled trial. Ann Surg 2019;269:2–9. 30080726 10.1097/SLA.0000000000002979

[11] Asbun HJ Moekotte AL Vissers FL . The Miami international evidence-based guidelines on minimally invasive pancreas resection. Ann Surg 2020;271:1–14. 31567509 10.1097/SLA.0000000000003590

[12] Cook JA McCulloch P Blazeby JM . IDEAL framework for surgical innovation 3: randomised controlled trials in the assessment stage and evaluations in the long term study stage. BMJ 2013;346:f2820. 23778425 10.1136/bmj.f2820 PMC3685513

[13] Ergina PL Barkun JS McCulloch P . IDEAL framework for surgical innovation 2: observational studies in the exploration and assessment stages. BMJ 2013;346:f3011. 23778426 10.1136/bmj.f3011 PMC3685514

[14] McCulloch P Cook JA Altman DG . IDEAL framework for surgical innovation 1: the idea and development stages. BMJ 2013;346:f3012. 23778427 10.1136/bmj.f3012 PMC3685515

[15] McCulloch P Altman DG Campbell WB . No surgical innovation without evaluation: the IDEAL recommendations. Lancet 2009;374:1105–1112. 19782876 10.1016/S0140-6736(09)61116-8

[16] Adam MA Choudhury K Dinan MA . Minimally invasive versus open pancreaticoduodenectomy for cancer: practice patterns and short-term outcomes among 7061 patients. Ann Surg 2015;262:372–377. 26158612 10.1097/SLA.0000000000001055

[17] Sharpe SM Talamonti MS Wang CE . Early national experience with laparoscopic pancreaticoduodenectomy for ductal adenocarcinoma: a comparison of laparoscopic pancreaticoduodenectomy and open pancreaticoduodenectomy from the National Cancer Data Base. J Am Coll Surg 2015;221:175–184. 26095569 10.1016/j.jamcollsurg.2015.04.021

[18] de Rooij T Cipriani F Rawashdeh M . Single-surgeon learning curve in 111 laparoscopic distal pancreatectomies: does operative time tell the whole story? J Am Coll Surg 2017;224:826–32.e1. 28126547 10.1016/j.jamcollsurg.2017.01.023

[19] Lof S Moekotte AL Al-Sarireh B . Multicentre observational cohort study of implementation and outcomes of laparoscopic distal pancreatectomy. Br J Surg 2019;106:1657–1665. 31454072 10.1002/bjs.11292

[20] Sahakyan MA Rosok BI Tholfsen T . Implementation and training with laparoscopic distal pancreatectomy: 23-year experience from a high-volume center. Surg Endosc 2022;36:468–479. 33534075 10.1007/s00464-021-08306-3 PMC8741682

[21] Nachmany I Pencovich N Ben-Yehuda A . Laparoscopic distal pancreatectomy: learning curve and experience in a tertiary center. J Laparoendosc Adv Surg Tech A 2016;26:470–474. 27149307 10.1089/lap.2016.0098

[22] Ricci C Casadei R Buscemi S . Laparoscopic distal pancreatectomy: what factors are related to the learning curve? Surg Today 2015;45:50–56. 24610347 10.1007/s00595-014-0872-x

[23] Mathew G Agha R . STROCSS 2021: strengthening the reporting of cohort, cross-sectional and case–control studies in surgery. Int J Surg 2021;96:106165. 34774726 10.1016/j.ijsu.2021.106165

[24] Gumbs AA Hilal MA Croner R . The initiation, standardization and proficiency (ISP) phases of the learning curve for minimally invasive liver resection: comparison of a fellowship-trained surgeon with the pioneers and early adopters. Surg Endosc 2021;35:5268–78. 33174100 10.1007/s00464-020-08122-1

[25] Ament R . Origin of the ASA classification. Anesthesiology 1979;51:179. 10.1097/00000542-197908000-00023 453623

[26] Bassi C Marchegiani G Dervenis C . The 2016 update of the International Study Group (ISGPS) definition and grading of postoperative pancreatic fistula: 11 years after. Surgery 2017;161:584–591. 28040257 10.1016/j.surg.2016.11.014

[27] Dindo D Demartines N Clavien PA . Classification of surgical complications: a new proposal with evaluation in a cohort of 6336 patients and results of a survey. Ann Surg 2004;240:205–213. 15273542 10.1097/01.sla.0000133083.54934.ae PMC1360123

[28] Montagnini AL Rosok BI Asbun HJ . Standardizing terminology for minimally invasive pancreatic resection. HPB (Oxford) 2017;19:182–189. 28317657 10.1016/j.hpb.2017.01.006

[29] Kimura W Inoue T Futakawa N . Spleen-preserving distal pancreatectomy with conservation of the splenic artery and vein. Surgery 1996;120:885–890. 8909526 10.1016/s0039-6060(96)80099-7

[30] Warshaw AL . Conservation of the spleen with distal pancreatectomy. Arch Surg 1988;123:550–553. 3358679 10.1001/archsurg.1988.01400290032004

[31] Müller PC Kuemmerli C Cizmic A . Learning curves in open, laparoscopic, and robotic pancreatic surgery: a systematic review and proposal of a standardization. Ann Surg Open 2022;3:e111. 10.1097/AS9.0000000000000111 PMC10431463 37600094

[32] Grigg OA Farewell VT Spiegelhalter DJ . Use of risk-adjusted CUSUM and RSPRT charts for monitoring in medical contexts. Stat Methods Med Res 2003;12:147–170. 12665208 10.1177/096228020301200205

[33] Steiner SH Cook RJ Farewell VT . Monitoring surgical performance using risk-adjusted cumulative sum charts. Biostatistics 2000;1:441–452. 12933566 10.1093/biostatistics/1.4.441

[34] Halls MC Alseidi A Berardi G . A comparison of the learning curves of laparoscopic liver surgeons in differing stages of the IDEAL paradigm of surgical innovation: standing on the shoulders of pioneers. Ann Surg 2019;269:221–228. 30080729 10.1097/SLA.0000000000002996

[35] van den Broek BLJ Zwart MJW Bonsing BA . Video grading of pancreatic anastomoses during robotic pancreatoduodenectomy to assess both learning curve and the risk of pancreatic fistula – a post hoc analysis of the LAELAPS-3 Training Program. Ann Surg 2023. doi: 10.1097/SLA.0000000000005796. Epub ahead of print. PMC10549894 36727842

[36] Zwart MJW Nota CLM de Rooij T . Outcomes of a multicenter training program in robotic pancreatoduodenectomy (LAELAPS-3). Ann Surg 2022;276:e886–e895. 33534227 10.1097/SLA.0000000000004783

[37] Braga M Ridolfi C Balzano G . Learning curve for laparoscopic distal pancreatectomy in a high-volume hospital. Updates Surg 2012;64:179–183. 22763577 10.1007/s13304-012-0163-2

