# Supplementary material for: Learning curves in laparoscopic distal pancreatectomy: a different experience for each generation
Source: Int J Surg. 2023 May 5;109(6):1648–55. doi: 10.1097/JS9.0000000000000408 (PMC10389345; doi:10.1097/JS9.0000000000000408)
Supplement: Supplementary file 1 [file js9-109-1648-s001.docx]

**SUPPLEMENTARY TABLES**

**SUPPLEMENTARY TABLE 1.** Risk-adjusted regression model of operative time using uni – and multivariable regression analyses

|  | **Univariable analysis** | | **Multivariable analysis** | |
| --- | --- | --- | --- | --- |
| **Variable** | **Standardized Beta (95% CI)** | ***p*** | **Standardized Beta (95% CI)** | ***p*** |
| Male | 20.577 (4.640 - 36.514) | **0.011** | 22.080 (6.294 - 37.866) | **0.006** |
| Age > 65 years | -5.496 (-21.565 - 10.572) | 0.502 | Removed step 1 |  |
| ASA III-IV | -21.567 (43.882 - 0.688) | 0.057 | -27.286 (-49.510 - -5.061) | **0.016** |
| BMI >30 kg/m^2^ | 5.849 (-20.039 - 31.737) | 0.657 | Removed step 2 |  |
| Multivisceral resection | 42.396 (11.532 - 73.460) | **0.007** | 44.643 (13.994 – 75.343) | **0.004** |
| PDAC | 20.539 (-2.732 - 43.810) | 0.084 | Removed step 5 |  |
| Size of lesion >5 cm | 8.546 (-11.555 - 28.648) | 0.404 | Removed step 3 |  |
| Splenectomy | -9.723 (-28.410 – 8.964) | 0.307 | Removed step 4 |  |

*Abbreviations: ASA= American Society of Anesthesiologists, BMI= Body Mass Index, kg/m^2^= kilogram per square meter, PDAC= pancreatic ductal adenocarcinoma, cm= centimeter, CI= confidence interval*

**SUPPLEMENTARY TABLE 2.** Risk-adjusted regression model of major complications using uni – and multivariable regression analyses

|  | **Univariable analysis** | | **Multivariable analysis** | |
| --- | --- | --- | --- | --- |
| **Variable** | **OR (95% CI)** | ***p*** | **OR (95% CI)** | ***p*** |
| Male | 0.782 (0.527-1.161) | 0.223 | Removed step 5 |  |
| Age > 65 years | 1.185 (0.810 - 1.732) | 0.382 | Removed step 7 |  |
| ASA III-IV | 1.092 (0.645-1.848) | 0.743 | Removed step 2 |  |
| BMI >30 kg/m^2^ | 1.902 (1.132-3.195) | **0.015** | 1.902 (1.132-3.195) | **0.015** |
| Multivisceral resection | 1.110 (0.543-2.269) | 0.775 | Removed step 1 |  |
| PDAC | 1.255 (0.738-2.135) | 0.402 | Removed step 6 |  |
| Size of lesion >5 cm | 1.146 (0.720 - 1.823) | 0.566 | Removed step 4 |  |
| Splenectomy | 0.956 (0.635 – 1.441) | 0.831 | Removed step 3 |  |

*Abbreviations: ASA= American Society of Anesthesiologists, BMI= Body Mass Index, kg/m^2^= kilogram per square meter, PDAC= pancreatic ductal adenocarcinoma, cm= centimeter, OR= odds ratio, CI= confidence interval*

**SUPPLEMENTARY FIGURES**

**SUPPLEMENTARY FIGURE 1.** **Detailed RA-CUSUM phase-1 feasibility learning curves for operative time; (a) among ‘trained’ surgeons, (b) among ‘self-taught’ surgeons.** *The point of maximum curvature of the polynomial curve shows the inflection point of the first phase of the learning curve, also demonstrated by the dotted reference lines (means) on the X – and Y-axis.*

**
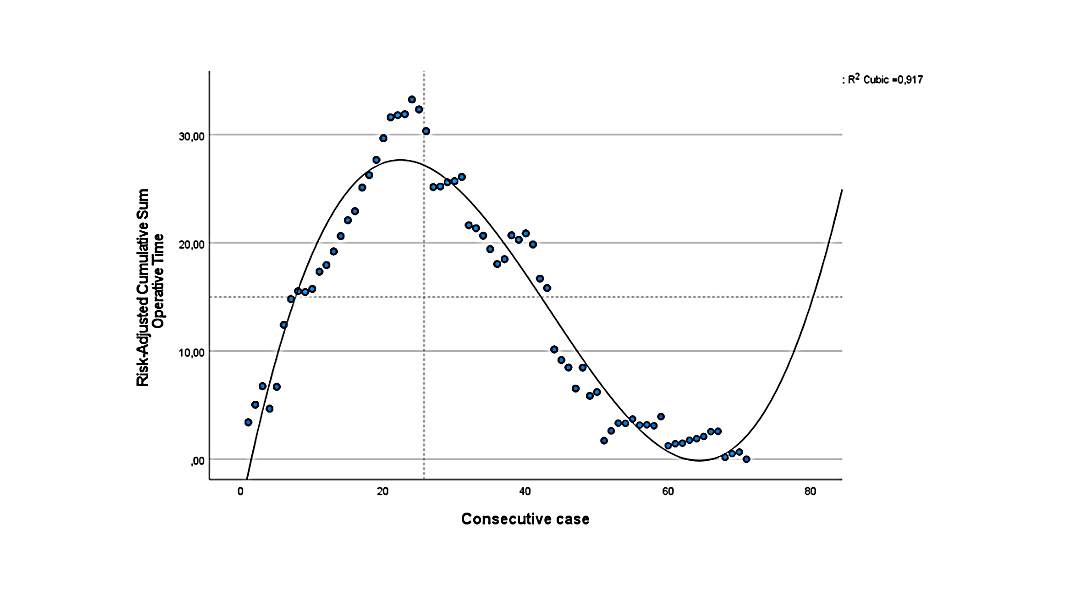

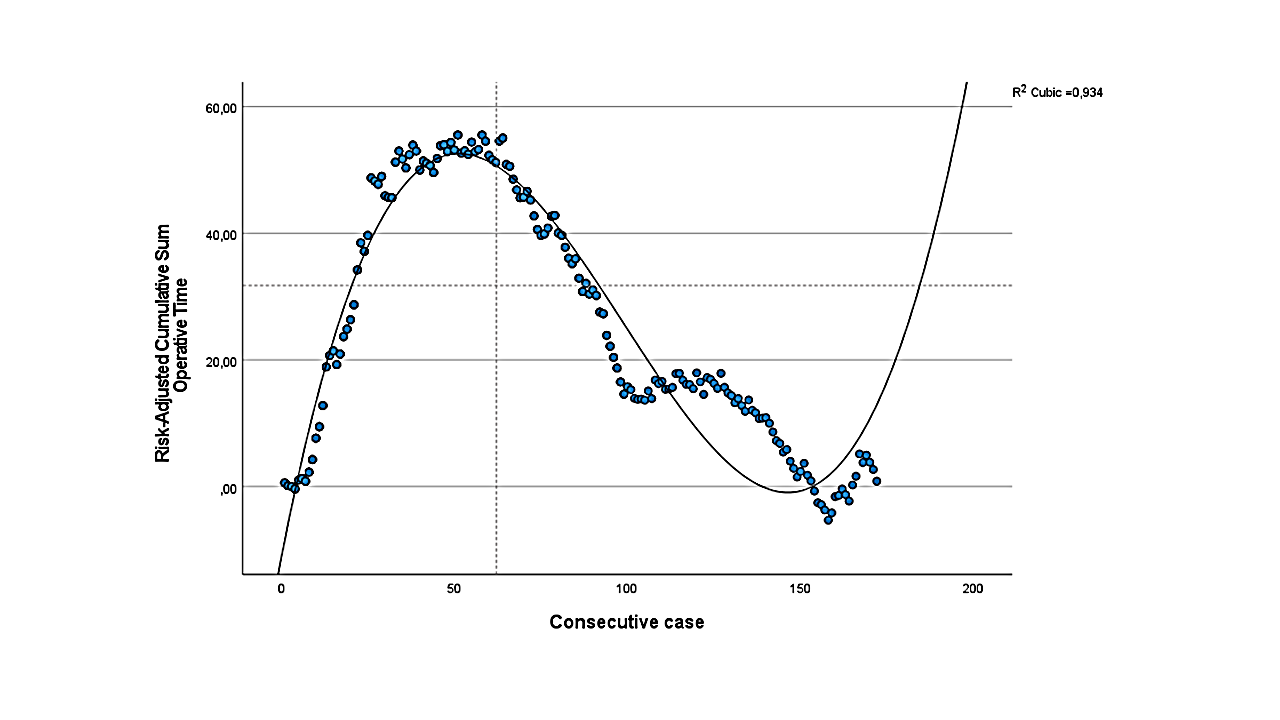
** **A B**

**SUPPLEMENTARY FIGURE 2.** **Detailed RA-CUSUM phase-2 proficiency learning curves for major complications; (a) among ‘trained’ surgeons, (b) among ‘self-taught’ surgeons.** *The second crossing point of two polynomial curves show the inflection point of the second phase of the learning curve, also demonstrated by the dotted reference lines (means) on the X – and Y-axis.*


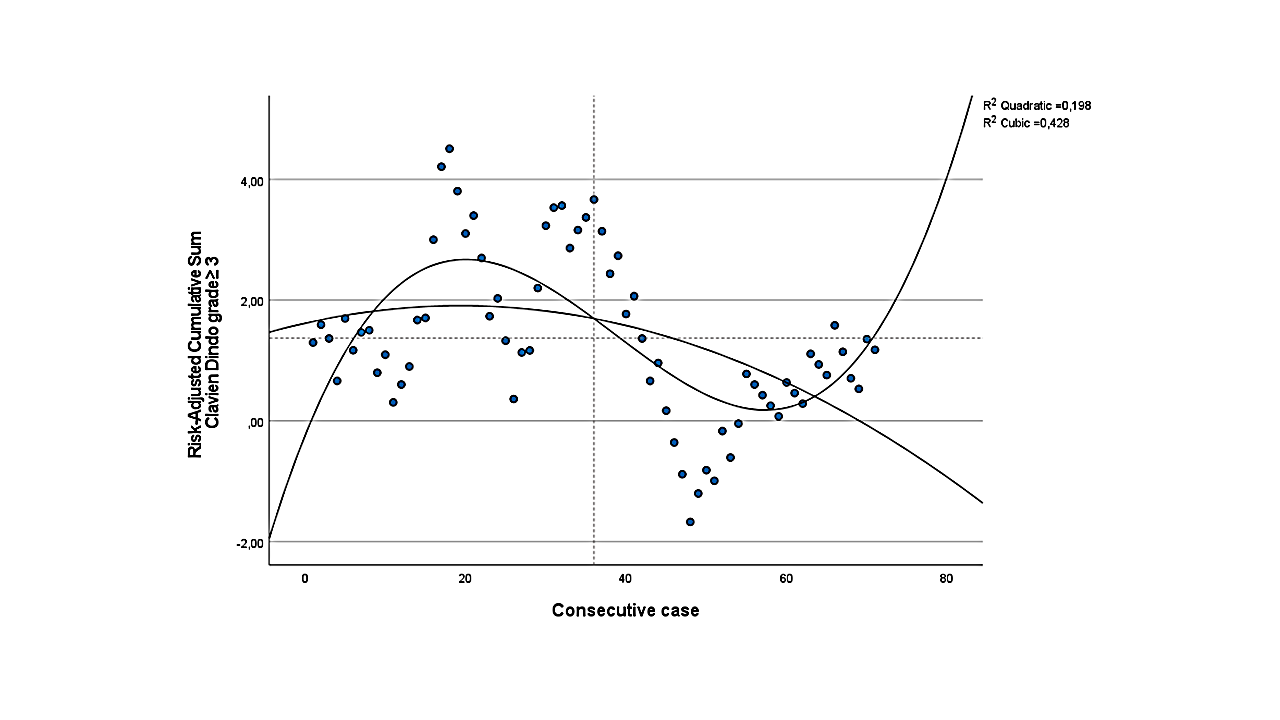


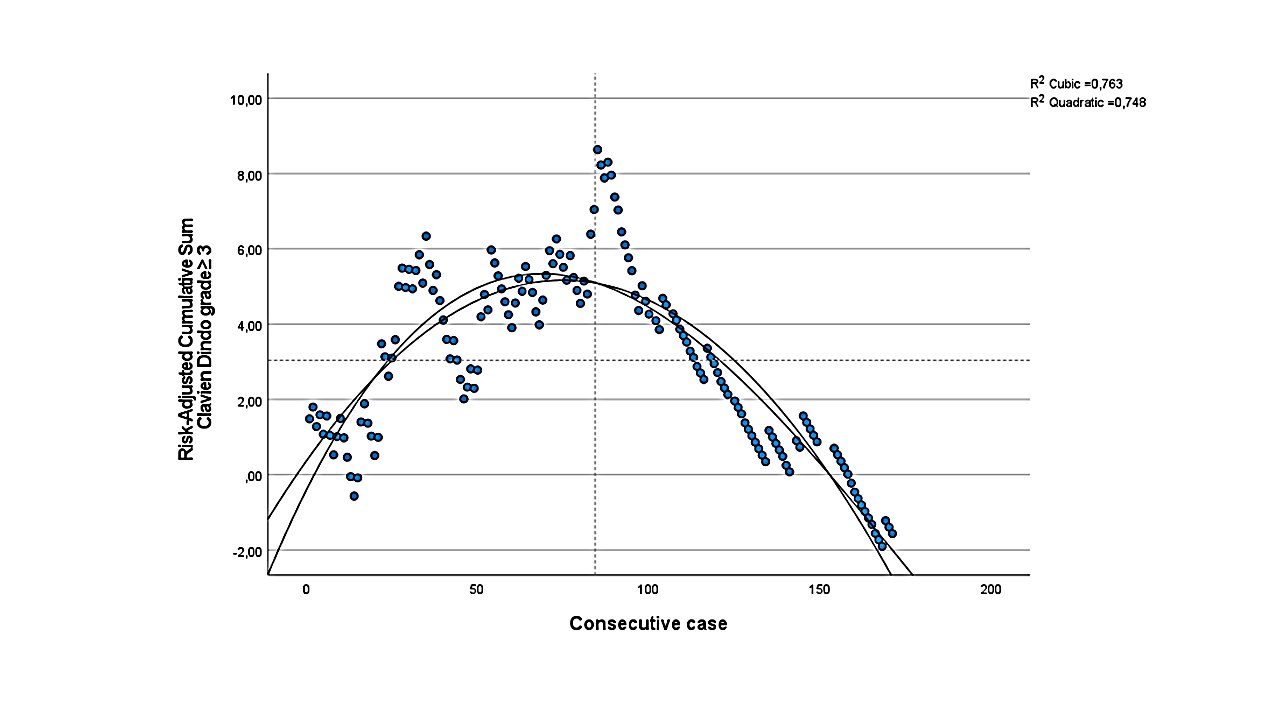
 **A B**
